# Magnetic moment impact on spin-dependent Seebeck coefficient of ferromagnetic thin films

**DOI:** 10.1038/s41598-022-26993-3

**Published:** 2023-01-04

**Authors:** Alain Portavoce, Elie Assaf, Maxime Bertoglio, Dario Narducci, Sylvain Bertaina

**Affiliations:** 1 IM2NP, Aix-Marseille University/CNRS, Faculté des Sciences de Saint-Jérôme case 142, 13397 Marseille, France; 2 Department of Materials Science, University of Milano-Bicocca, Via R. Cozzi 55, 20125 Milan, Italy

**Keywords:** Magnetic properties and materials, Thermoelectrics

## Abstract

Magnetic materials may be engineered to produce thermoelectric materials using spin-related effects. However, clear understanding of localized magnetic moments (*µ*_*I*_), free carriers, and Seebeck coefficient (*S*) interrelations is mandatory for efficient material design. In this work, we investigate *µ*_*I*_ influence on the spin-dependent *S* of model ferromagnetic thin films, allowing *µ*_*I*_ thermal fluctuations, ordering, and density variation influence to be independently investigated. *µ*_*I*_ influence on free carrier polarization is found to be of highest importance on *S*: efficient coupling of free carrier spin and localized magnetic moment promotes the increase of *S*, while spin-dependent relaxation time difference between the two spin-dependent conduction channels leads to *S* decrease. Our observations support new routes for thermoelectric material design based on spin-related effects in ferromagnetic materials.

## Introduction

**Figure 1:**
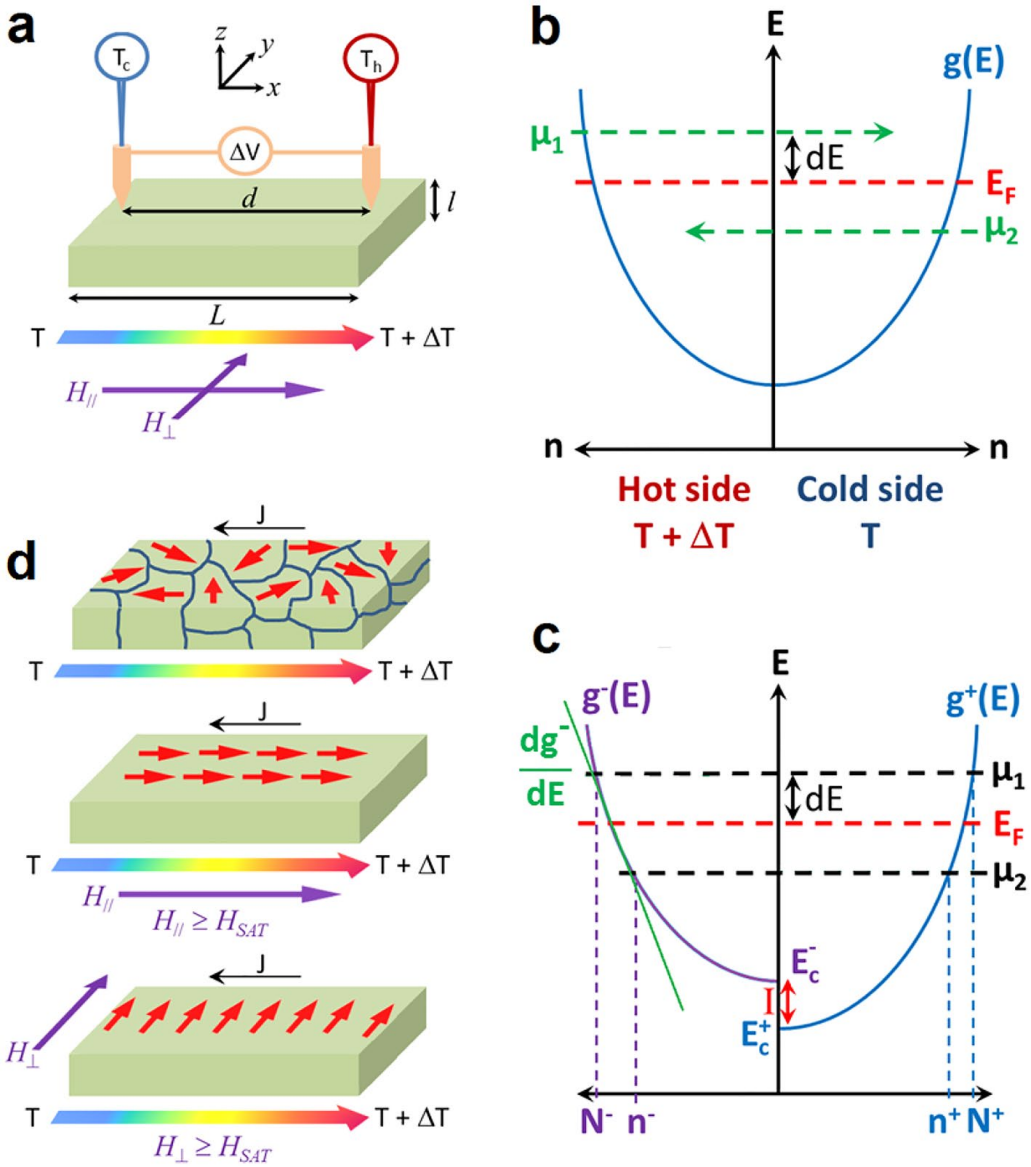
Schematics of *S* measurements in FM thin films and schematics of electronic DOS in PM and FM metallic materials under temperature gradient. (**a**) *S* measurement principle. (**b**) DOS of a PM metallic-type material under temperature gradient. (**c**) DOS of a FM metallic-type material under temperature gradient. (**d**) ***µ***_***I***_ distribution in a FM thin film under temperature gradient without magnetic field, or at saturation with a parallel (*H*_//_) or perpendicular (*H*_⊥_) magnetic field.

Energy saving is an important technological topic with many awaiting challenges, in particular in the case of the development of mobile technologies integrating a growing number of functionalities. Accompanying low-energy consumption device and high-power density battery development, energy harvesting technologies aim also at increasing portable electronic device autonomy^1–3^. Temperature gradients being usually present in microelectronic setups, integration of thermoelectric (TE) devices is currently explored^1,3^. TE solutions based on thin films compatible with the complementary-metal–oxide–semiconductor technology (CMOS) have already been proposed supporting TE device integration^4,5^. However, TE solutions for microelectronic applications need to operate close to room temperature (RT), TE efficiency being mainly dependent on intrinsic material properties, such as thermal conductivity (*κ*), electrical conductivity (*σ*), and Seebeck coefficient (*S*), TE technology improvement requires either the development of new materials, or to develop engineering methods allowing TE properties of current materials to be improved. Concurrent with *κ* engineering, recent band engineering solutions were proposed to increase the TE power factor *PF* = *S*^2^*σ*^6^, such as modulation doping^7,8^, resonance levels^9,10^, energy filtering^11–13^, and quantum confinement^14^. The thermopower *S* is related to the Peltier coefficient П such as *S* = П/*T*, П corresponding to the energy carried by the mobile charge carriers in the material per unit of charge. Figure 1a presents the method used for the measurement of *S* = Δ*V*/Δ*T*. Semiconductor materials are extensively studied^15–18^, as a same semiconductor can be used as *n*-type or *p*-type TE material depending on doping, and they allow substantial band engineering. However, interest of spin effects on material TE properties is growing, and investigations on magnetic material potential for TE applications has considerably raised. In particular, the spin-Seebeck effect offers new routes for converting waste heat to electric power^19^. Based on spin transport, spin-Seebeck was demonstrated in different types of ferromagnetic materials: metallic^20^, semiconductor^21^, and insulator^22^. Investigations of magnetism influence on material TE performance have been reported^23^, and original ferromagnetic materials^23–25^ and spintronic structures^24,26^ have been proposed for TE energy harvesting. Spin effects on the conventional Seebeck coefficient were shown to provide interesting ways of *S* engineering, based on charge carrier interactions with localized magnetic moments through the magnon-drag effect^27^ or the spin fluctuation effect^28^, for example. Thus, spin effect engineering in ferromagnetic (FM) materials should be considered as a possible way of obtaining improved TE properties, and interactions of mobile charge carriers with localized magnetic moments should be thoroughly investigated.

In this work, the influence of interactions between localized magnetic moments and spin-polarized free electrons on the spin-dependent Seebeck coefficient is investigated in FM thin films exhibiting metallic conduction and low charge carrier density. In order to separate as much as possible the effects of magnetic moment fluctuation, ordering, and density, the investigations focus on two FM germanides Mn_5_Ge_3_^29–34^ and MnCoGe^35–43^, with magnetization (***M***) depending on a single element (almost single magnetic moment ***µ***_***I***_ carried by Mn ions, Supplementary Fig. S1), and exhibiting Curie temperatures (*T*_*c*_) close to RT, allowing *S* measurements and *M* variations at *T*_*c*_ to be compared in the same temperature range. The film geometry allows ***µ***_***I***_ degree of freedom to be reduced, as the moments are forced to be aligned in the film plane^42,44^.

### Spin-dependent Seebeck coefficient

In the following, the term spin-dependent Seebeck coefficient is used to designate the regular Seebeck coefficient measured in FM materials below the Curie temperature. For paramagnetic (PM) metallic materials in a temperature gradient, *S* depends on the asymmetry of a single density of states (DOS) *g*(*E*) close to the average Fermi level (*E*_*F*_) corresponding to the considered temperature gradient (Fig. 1b). The average electrochemical potential (*µ*_1_) of electrons above *E*_*F*_ promotes the diffusion of electrons towards the sample’s cold side, while the average electrochemical potential (*µ*_2_) of electrons below *E*_*F*_ promotes the diffusion of electrons towards the sample’s hot side (Fig. 1b), due to the difference of electronic state filling between the two sides.

The net electron flux is given by the difference between these two fluxes, and leads to the accumulation of electrons at one side of the sample and immobile matrix ions at the other side of the sample, building the potential difference Δ*V* =  − (*µ*_1_ − *µ*_2_)/*e* (*e* is the elementary charge). The Seebeck coefficient can be expressed as:

1 $$S = - {\triangle\mu /eT,}$$

with Δ*µ* = *µ*_1_ − *µ*_2_ (Fig. 1b). However, *S* depends on the asymmetry of the two DOS *g*^+^(*E*) and *g*^-^(*E*) of respectively majority-spin and minority-spin electrons (Fig. 1c), leading to two separate electronic currents in the case of FM materials: (i) the current of majority-spin electrons related to *g*^+^(*E*) and the electron densities *N*^+^ above *E*_*F*_ and *n*^+^ below *E*_*F*_, as well as (ii) the current of minority-spin electrons related to *g*^−^(*E*) and the electron densities *N*^−^ above *E*_*F*_ and *n*^*−*^ below *E*_*F*_. In this case Δ*µ* = Δ*µ*^+^  + Δ*µ*^*−*^ in Eq. 1, with Δ*µ*^+^  = *µ*_1_^+^  − *µ*_2_^+^ and Δ*µ*^*−*^  = *µ*_1_^−^ − *µ*_2_^−^. Considering the simplified model presented in Fig. 1c, *S* can be expressed as

2 $$S=-\frac{1}{2eT}\frac{d\varepsilon }{n}\left[{\left(\frac{d{g}^{+}}{d\epsilon }\right)}_{{E}_{F}}-{\left(\frac{d{g}^{-}}{d\epsilon }\right)}_{{E}_{F}}\right]\left({\Delta E}_{C}+\alpha \Delta {E}_{eI}+\Delta R{k}_{B}T\right),$$

considering that the FM material of interest reports a relatively low density of carriers^45^. The carrier density in the considered Mn_5_Ge_3_ and MnCoGe films is respectively 1.6 × 10^20^ cm^−3^ and 1.7 × 10^18^ cm^−3^ according to RT Hall effect measurements. *dε* is the energy variation around *E*_*F*_ involved with the temperature gradient, and *n* = *n*^+^  + *n*^*−*^ is the entire number of electronic state close to *E*_*F*_ (below *E*_*F*_ according to Fig. 1c). Δ*E*_*C*_ = *E*_*C*_^*−*^ − *E*_*C*_^+^ = *I* in Fig. 1c corresponds to the energy difference between the bottom energies *E*_*C*_^*−*^ and *E*_*C*_^+^ of the conduction bands of the minority-spin and majority-spin electrons, respectively. Δ*E*_*eI*_ = *E*_*eI*_^*−*^ − *E*_*eI*_^+^ with *E*_*eI*_^*j*^ the average energy related to the polarization of the magnetic moment of conduction electrons of spin polarization *j* by the localized magnetic moments. This parameter does not consider macroscopic effects, such as those due to magnetic domains’ walls for example. Thus, the parameter *α* is added in order to take into account a statistical efficiency of free electron polarization. Δ*R* = *R*^+^ − *R*^*−*^ with *R*^*j*^ a dimensionless constant related to the average energy of electrons of spin polarization *j* depending on scattering mechanisms^46,47^. Considering that at given *T* (*dg*^+^/*dε*)_*EF*_ = Ω^+^ and (*dg*^−^/*dε*)_*EF*_ = Ω^−^, one can define a parameter *β*(*T*) = *dε*/*n* (Ω^+^ − Ω^−^). In this case

3 $$S=-\frac{\beta }{2eT}\left({\Delta E}_{C}+\alpha \Delta {E}_{eI}+\Delta R{k}_{B}T\right).$$

### Localized magnetic moment fluctuation

**Figure 2:**
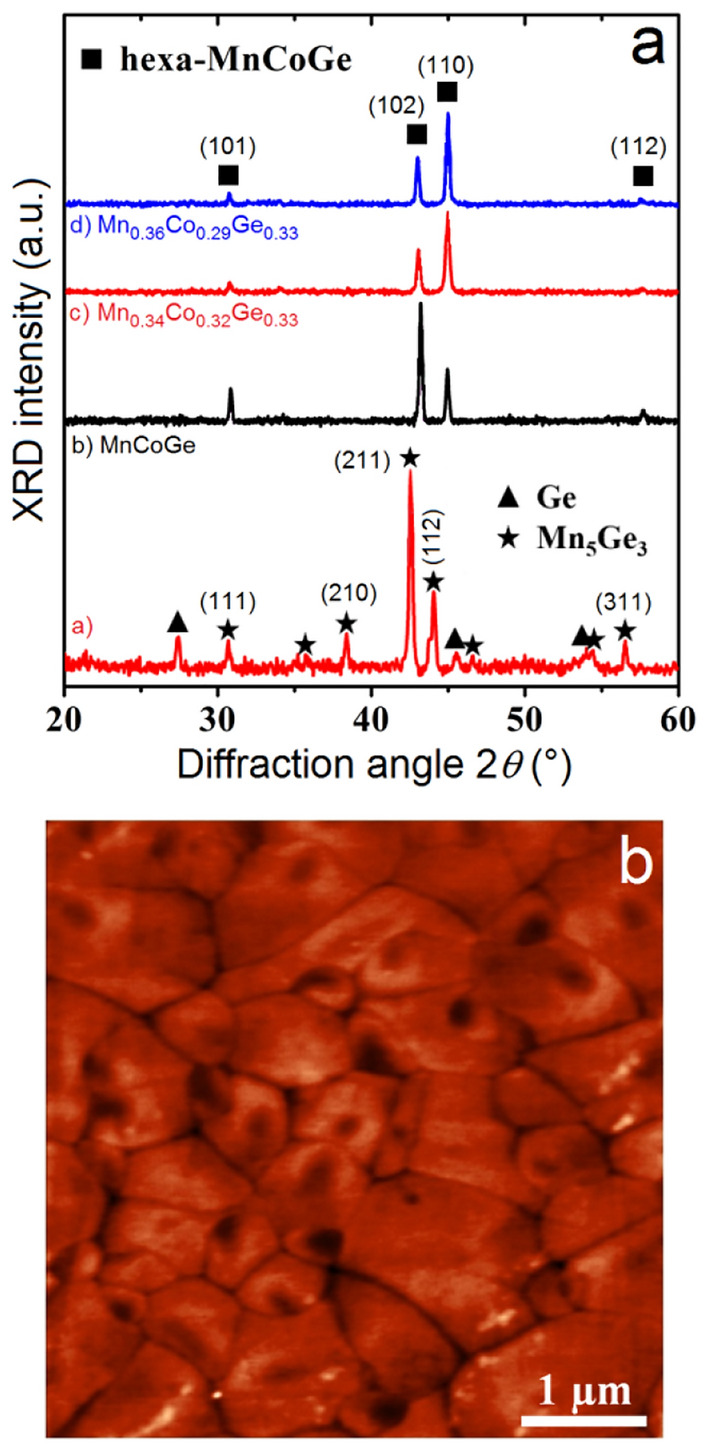
Microstructures of the 50 nm-thick Mn_5_Ge_3_ film and of the 150 nm-thick Mn_*x*_Co_*y*_Ge_1−*x*−*y*_ films. (**a**) X-ray diffractograms measured on Mn_5_Ge_3_ and Mn_*x*_Co_*y*_Ge_1−*x*−*y*_ films. (**b**) AFM measurements performed on the film MnCoGe.

**Figure 3:**
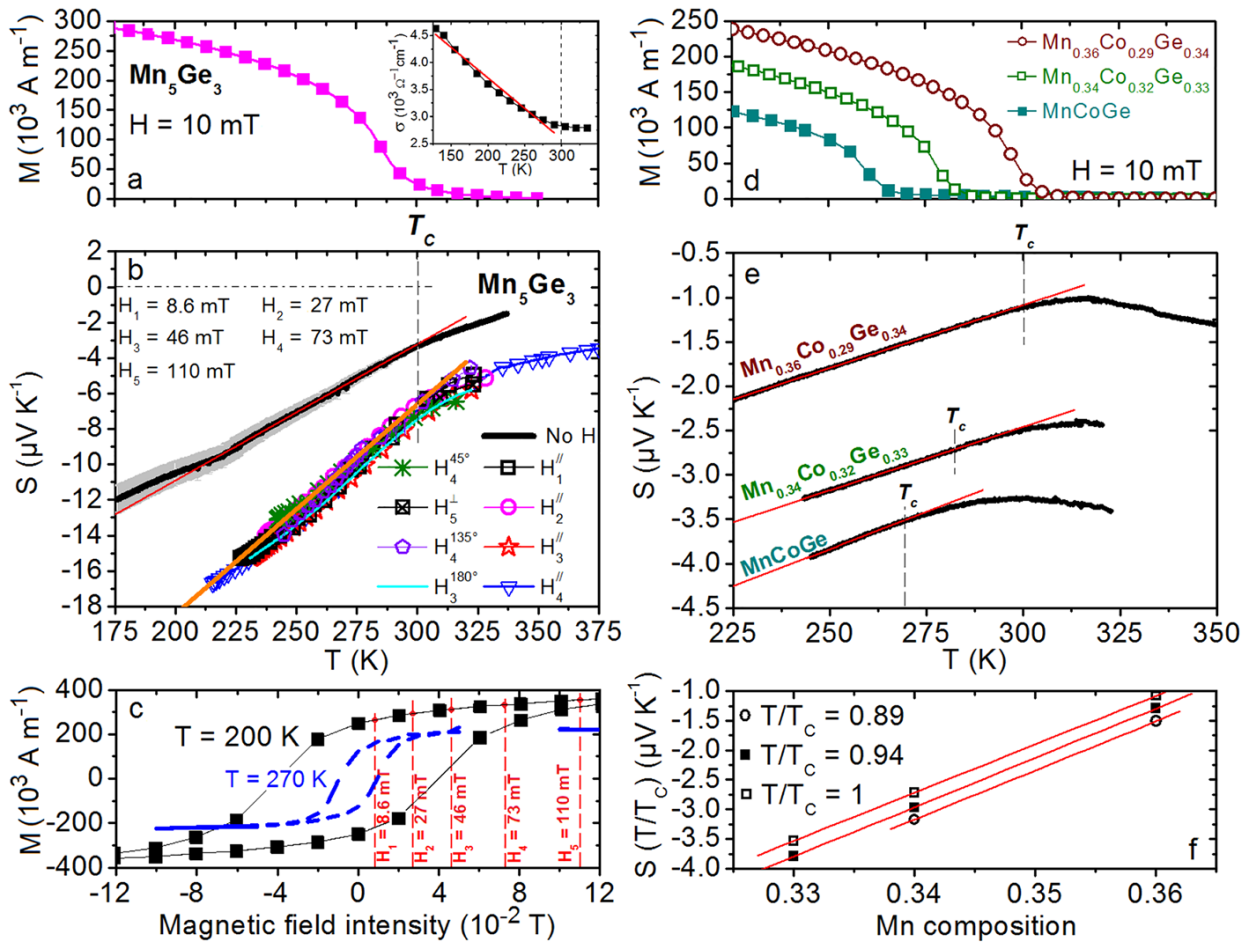
Magnetization (*M*) and Seebeck coefficient (*S*) measurements performed on Mn_5_Ge_3_ and MnCoGe thin films. *M* (**a**) and *S* (**b**) of a 50 nm-thick Mn_5_Ge_3_ film as a function of temperature. The inset in (**a**) presents the electrical conductivity of the film versus temperature. (**c**) *M* measured at 200 K (black solid squares) and 270 K (blue dashed line) on a same Mn_5_Ge_3_ film as a function of in-plane magnetic field intensity. *M* (**d**) and *S* (**e**) of 150 nm-thick Mn_*x*_Co_*y*_Ge_1−*x*−*y*_ films as a function of temperature. (**f**) *S*(*T*/*T*_*c*_) of same Mn_*x*_Co_*y*_Ge_1−*x*−*y*_ films plotted as a function of Mn composition *x*.

The diffractogram (a) in Fig. 2a was acquired on the Mn_5_Ge_3_ film (Supplementary Fig. S1a). The film is polycrystalline and the Scherrer equation^48^ applied to the most intense diffraction peak Mn_5_Ge_3_(211) indicates that the layer is composed of columnar grains with a thickness ~ 49 ± 5 nm. Grains exhibit an average lateral size ~ 1 µm and the root mean square (RMS) surface roughness of the film is ~ 1.8 nm according to AFM measurements^44^. Figure 3a shows the variation of the Mn_5_Ge_3_ film magnetization versus temperature in the temperature range 175 ≤ *T* ≤ 350 K. *M* decreases as temperature increases up to the Curie temperature of the FM/PM transition at *T*_*c*_ = 297 K. The Curie temperature corresponds to the carbon-free Mn_5_Ge_3_ compound^49^. The electrical conductivity variation of the sample versus *T* is presented in the inset. The conductivity decreases almost linearly when the temperature increases from 150 K up to *T*_*c*_. Figure 3b displays the variations of the spin-dependent *S* of the layer in the same temperature range. The black line corresponds to measurements performed without external magnetic field. The Mn_5_Ge_3_ film was not exposed to any magnetic field before Seebeck measurements. Consequently, the net magnetization is essentially zero in these conditions. The gray envelop around the data corresponds to the maximum measurement error observed on *S* in this study. This error considers both elaboration and Seebeck measurement reproducibility. It is only shown on this measurement for clarity.

Mn_5_Ge_3_ is *n*-type as *S* < 0. *S* decreases as temperature below the FM/PM transition increases, following a linear behavior from *T* ~ 215 K to *T* = *T*_*c*_. Remarkably, *S* and *σ* are found to concurrently increase as temperature decreases, leading to a *PF* increase > 1700% from 300 to 175 K. Without external field, the Mn_5_Ge_3_ film exhibits weak magnetization due to moment disorder and fluctuations, in particular at RT close to *T*_*c*_. However, magnetic domains (~ 0.65 × 1.5 µm^2^) as depicted in Fig. 1d can still be observed in the film at that temperature by magnetic force microscopy^44^.

When the temperature increases towards *T*_*c*_, the fluctuation of localized moments increases and the distribution of the ***µ***_***I***_ orientations increases: the size of the magnetic domains decreases and the density of magnetic domain walls increases. The magnetization of the film decreases at the same time, in a similar way as observed in Fig. 3a. *S* decreases linearly during this process (Fig. 3b). Constant parameters *A* and *B* were determined experimentally from the measurements presented in Fig. 3b, considering^47^

4 $$S=-\frac{1}{eT}\left(A+B{k}_{B}T\right).$$

They are reported in Table 1. Assuming that Δ*E*_*C*_ is negligible for Mn_5_Ge_3_^31,34^ and combining Eqs. (3) and (4) one obtains *A* = *βα*Δ*E*_*eI*_/2 and *B* = *β*Δ*R*/2. Consequently, the linear behavior of *S* versus *T* suggests that the localized moment fluctuation effect occurs at constant *β*, constant *α*Δ*E*_*eI*_, and constant Δ*R*, with *βα*Δ*E*_*eI*_ = 0.01 eV and *β*Δ*R* ~  − 1/3. *β* depends on the DOS of spin-up and spin-down electrons (Fig. 1c) and should a priori vary with temperature. However, the energy variation related to a temperature change from 200 to 300 K is only ~ 8.62 × 10^−3^ eV, which may explain that the parameter *β* is found to be almost constant in our temperature range of investigation. Δ*E*_*eI*_ is related to the energy gain involved with the polarization of free electrons by the magnetic moments localized on Mn ions. Considering that electron spin-up *s*_*e*_^+^ =  + ½ and electron spin-down *s*_*e*_^*−*^ =  − ½, Δ*E*_*eI*_ = ½ (*J*_*eI*_^+^ + *J*_*eI*_^*−*^)*µ*_*I*_ cos*θ*, with *J*_*eI*_^*j*^ the exchange parameter of spin-up electrons (*j* = ‘ + ’) or of spin-down electrons (*j* = ‘ − ’), and *θ* the angle between the localized moment and the electron spin. *J*_*eI*_^*j*^ and *µ*_*I*_ are expected to be independent of *T* in this model, and *θ* can be fixed to *θ* = 0 since Δ*E*_*eI*_ corresponds to an average energy. Thus, Δ*E*_*eI*_ should indeed be independent of *T*. *α* is also found to be independent of *µ*_*I*_ fluctuations with *T* close to *T*_*c*_ in Mn_5_Ge_3_. Δ*R* is related to the spin-dependence of free carrier scattering mechanisms. Indeed, the free carrier relaxation time *τ* is expected to be spin-dependent in FM materials, which is responsible for free carrier polarization *P* = (*γ* − 1)/(*γ* + 1) with *γ* = *τ*^+^/*τ*^−^. In our case, both Mn ions^50^ and magnetic domain walls can act as spin-dependent scattering centers^51,52^.

**Table 1:** Parameters *A* and *B* in Eq. (4) determined experimentally from *S* measurements presented in Fig. 3b for Mn_5_Ge_3_ films and Fig. 3e for Mn_*x*_Co_*y*_Ge_1−*x*−*y*_ films.

		*A* (eV)	*B*
Mn_5_Ge_3_	*H* = 0	0.005	− 0.16
	*H* = *H*_*SAT*_	0.01	− 0.30
MnCoGe	*H* = 0	0.001	− 0.008
Mn_0.34_Co_0.32_Ge_0.33_	*H* = 0	0.001	− 0.011
Mn_0.36_Co_0.29_Ge_0.34_	*H* = 0	0.001	− 0.022

Thermal fluctuations of localized magnetic moments lead to the decrease of the spin-dependent *S* along with magnetization (and polarization). The increase of the product *βα*Δ*E*_*eI*_ promotes the increase of *S*, while the increase of the product *β*Δ*R* leads to a decrease of *S*, since *A* > 0 and *B* < 0 (Table 1).

### Magnetic moment ordering

Mn_5_Ge_3_ spin-dependent *S* variations versus temperature were also studied under an external magnetic field ***H*** applied in the film plane as shown in Fig. 1a. Figure 3b presents *S* variations versus temperature for four different magnetic field intensities *H*^//^ = 8.6 × 10^−3^, 27 × 10^−3^, 46 × 10^−3^, and 73 × 10^−3^ Tesla in the direction parallel to the temperature gradient (Fig. 1a), as well as *S* measurements performed under an external magnetic field either oriented at 45° (*H*_*4*_^45°^ = 73 × 10^−3^ T), 90° (*H*_*5*_^⊥^ = 110 × 10^−3^ T), 135° (*H*_*4*_^135°^ = 73 × 10^−3^ T), and 180° (*H*_*3*_^180°^ = 46 × 10^−3^ T) compared to the temperature gradient direction (Fig. 1a). *S* variations versus temperature in presence of ***H*** are again found to be linear up to the FM/PA transition. The external magnetic field ***H*** promotes the increase of *S* at constant *T*, but the in-plane field effect is independent of the field direction and of the field intensity. Figure 3c shows the magnetization variations of the Mn_5_Ge_3_ film versus in-plane magnetic field intensity measured at 270 K (blue dashed line) and 200 K (solid squares). According to the magnetic hysteresis loop, the film magnetization was at saturation for all the *S* measurements performed under external magnetic field (*H* = *H*_*SAT*_), which can explain ***H*** effect on *S* to be independent of ***H*** intensity and orientation, considering the influence of the external field to be related to localized moment ordering and magnetic domain wall density variations (Fig. 1d). Maximum polarization of conduction electrons being reached at magnetization saturation, one should consider *α* = 1 in this case, and *α* < 1 otherwise. Considering *α* = 1 for *S* measurements performed on the Mn_5_Ge_3_ films under external magnetic field (Fig. 3b and Table 1), we obtain *α* = ½ without magnetic field, which is coherent with the considered model. The product *β*Δ*E*_*eI*_ = 0.02 eV at magnetization saturation. For comparison, the coupling energy of the localized moments should be of the order of *k*_*B*_*T*_*c*_ = 0.026 eV (Fig. 3a). Comparing the values of *B* with and without external magnetic field (Table 1), the scattering parameter Δ*R*_*SAT*_ (*H* = *H*_*SAT*_) is found to be twice as Δ*R*_*0*_ (*H* = 0), with Δ*R*_*0*_/Δ*R*_*SAT*_ ~ ½ < 1. This means that the relaxation time difference Δ*τ* between minority- and majority-spin electrons increases under magnetic field, and is found to scale with the parameter *α* in Mn_5_Ge_3_. These results agree with a higher polarization of conduction electrons at magnetization saturation. The polarization of free carriers has two opposite effects on the spin-dependent *S*: localized moment ordering promotes (i) an increase of *A* = *βα*Δ*E*_*eI*_/2 due to a statistical increase of ***s***_***e***_ and ***µ***_***I***_ pairing, increasing *S*, and (ii) an increase of Δ*τ*, decreasing *S*. However, the global effect of localized magnetic moment ordering in FM Mn_5_Ge_3_ promotes the increase of *S* at given *T*. ***H*** effect agrees with thermal fluctuation effect, as in the two cases, the spin-dependent *S* increases with localized moment polarization. This behavior can be the signature of bipolar conduction, since in the case of a FM film containing a single moment ***µ***_***I***_, *M* = (*n*^+^ − *n*^*−*^)*µ*_*I*_ with *n*^+^ and *n*^*−*^ the concentrations of occupied states in each level (up and down).

### Magnetic moment density

**Figure 4:**
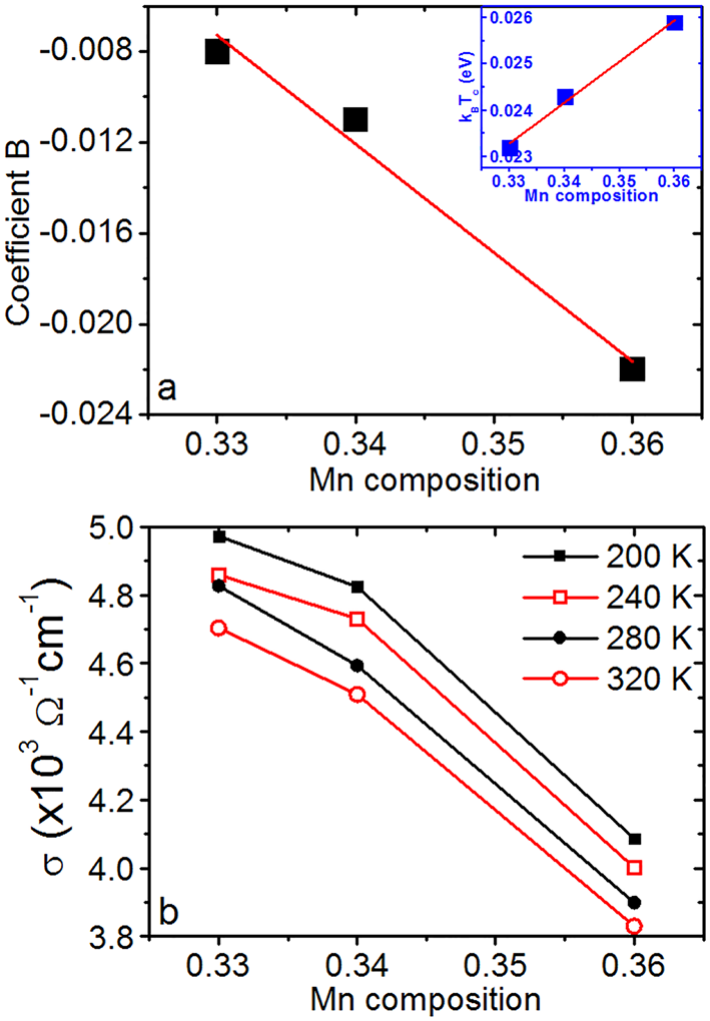
Electrical properties of the Mn_*x*_Co_*y*_Ge_1−*x*−*y*_ thin films. (**a**) electron-scattering-dependent parameter *B* (Eq. 4) as a function of *x*. The inset shows the *k*_*B*_*T*_*c*_ energy variation versus *x*. (**b**) Electrical conductivity as a function of *x*, measured at different temperatures.

Figure 2a presents three diffractograms (b), (c), and (d), acquired on three MnCoGe films of different Mn composition. The films contain only the hexagonal MnCoGe phase (Supplementary Fig. S1b). They are polycrystalline and composed of columnar grains with a thickness ~ 152 ± 5 nm. The RMS surface roughness is ~ 0.9 nm and the MnCoGe grains exhibit an average lateral size ~ 1.8 × 0.7 µm^2^ for the three films MnCoGe, Mn_0.34_Co_0.32_Ge_0.33_, and Mn_0.36_Co_0.29_Ge_0.34_ according to AFM measurements (Fig. 2b). Figure 3d shows the magnetization variations versus temperature of the three Mn_*x*_Co_*y*_Ge_1−*x*−*y*_ films. Magnetization and Curie temperature increase with *x*, which correspond to an increase of the density of localized moment ***µ***_***I***_ and to an increase of the exchange energy between ***µ***_***I***_, respectively. Figure 3e presents the variations versus temperature of the spin-dependent *S* of the same films. Similar to Mn_5_Ge_3_, *S* is negative (*n*-type) and decreases linearly as temperature increases up to *T*_*c*_ for the three films. The FM/PA transition is more easily detected in the case of MnCoGe films, especially for Mn_0.36_Co_0.29_Ge_0.34_, *S* increasing with temperature after *T*_*c*_. This change of behavior of *S* versus *T* is commonly observed in FM metals, *S* variations resulting from the bipolar effect of spin-up and spin-down electrons before *T*_*c*_ and *S* following the behavior of common metals after *T*_*c*_^53^. Furthermore, *S* is found to decrease as the Mn concentration increases in the MnCoGe films. The parameters *A* and *B* in Eq. (4) were determined from *S* measurements presented in Fig. 3e. They are displayed in Table 1. The slope of the linear function *S* = *f*(*T*) is independent of the Mn concentration with *A* = 0.001 eV for the three samples (Table 1), giving *βα*Δ*E*_*eI*_ = 0.002 eV if Δ*E*_*C*_ = 0 in Eq. (3). This result suggests that the 3 at% increase of Mn atoms in the compound MnCoGe does not involve a significant modification of the MnCoGe DOS close to *E*_*F*_ in our temperature range, supporting a constant parameter *β*, independent of Mn concentration. Furthermore, constant *α*Δ*E*_*eI*_ suggests also that the increase of exchange energy between localized moments ***µ***_***I***_ has no influence on the average coupling energy of the conduction electron spin ***s***_***e***_ with ***µ***_***I***_. Figure 3f presents *S* variations versus the Mn composition of Mn_*x*_Co_*y*_Ge_1−*x*−*y*_ films. *S* is inversely proportional to *x*. This behavior is the result of the linear decrease of the parameter *B* as *x* increases in Mn_*x*_Co_*y*_Ge_1−*x*−*y*_ films (Fig. 4a), suggesting an increase of Δ*τ* with Mn concentration (Tab. 1), which is in agreement with the electrical conductivity behavior of Mn_*x*_Co_*y*_Ge_1−*x*−*y*_ films versus *x*. Indeed, Fig. 4b shows that *σ* decreases as *x* increases. *σ* = *enµ*_*e*_ can decrease either due to a decrease of carrier density *n*, or due to a decrease of carrier mobility *µ*_*e*_.

However, *β* being independent of Mn concentration, the linear decrease of *S* versus *x* appears to be related to a decrease of *µ*_*e*_ = *eτ*/m^*^, due to a modification of the average scattering time *τ* in the two spin-dependent channels, resulting from a scattering effect associated with the additional Mn atoms. The increase of the magnetic moment density in MnCoGe have negligible effect on the exchange energy between conduction electron spin and localized ***µ***_***I***_, but promotes an increase of Δ*τ*, leading to the decrease of *S*.

Extra Mn ions can act as additional spin-dependent scattering centers^50^, supporting a stronger spin-dependent scattering, which should be accompanied with a higher polarization of free electrons^50^. However, the polarization increase is not observed on the slope of the linear function *S* = *f*(*T*), such as in the case of Mn_5_Ge_3_ films under external magnetic field (Fig. 3b). Δ*τ* could also be related to conduction electron bipolarity. Indeed, in the case of bipolar conduction, majority-spin electrons should diffuse towards the cold side (*n*-type *S*), while minority-spin electrons should diffuse towards the hot side, which could explain a difference of scattering conditions in the two channels independent of *α*. The decrease of *S* with the increase of Mn concentration should not be related to the corresponding polarization increase (Fig. 3d), since the spin-dependent *S* is observed to increase with magnetic polarization in both Mn_5_Ge_3_ (Fig. 3b) and Mn_*x*_Co_*y*_Ge_1−*x*−*y*_ (Fig. 3e). Instead, the decrease of *S* with the increase of *x* is related to the corresponding increase of the direct exchange energy *E*_*ex*_ =  − *J*_*ex *_***µ***_***I***_**.*µ***_***I***_ between Mn magnetic moments. Since ***µ***_***I***_ coupling vanishes at *T* = *T*_*c*_, *E*_*ex*_ ~ *k*_*B*_*T*_*c*_ can be assumed. The insert in Fig. 4a presents the variations of the product *k*_*B*_*T*_*c*_ versus *x*. *E*_*ex*_ is found to increase linearly with the Mn content. Consequently, free electron scattering in FM MnCoGe mainly involves coupled Mn magnetic moments, explaining the linear increase of the spin-dependent scattering difference (Δ*R* in Eq. 3) between the two spin-dependent channels with Mn composition by the linear increase of moments’ exchange energy.

### Outlook

The influence of thermal fluctuations, ordering, and density of localized magnetic moments on the spin-dependent Seebeck coefficient *S* has been investigated in ferromagnetic Mn_5_Ge_3_ and MnCoGe thin films. *S* is found to be mainly sensitive to the polarization of the conduction electrons, according to two opposite mechanisms: (i) the coupling of the spin vector of free carriers with the localized moments leads to higher *S*, while (ii) an increase of the scattering difference in the two spin-dependent conduction channels leads to lower *S*. In the case of Mn_5_Ge_3_ films under external magnetic field, the overall contribution of the two mechanisms leads to an increase of *S*. The thermal fluctuations of localized magnetic moments, as well as moment ordering using an external magnetic field show coherent effects, as in both cases *S* increases with localized moment polarization. The increase of Mn concentration in hexagonal MnCoGe leads to an increase of the magnetic moment density and of the localized moment exchange energy. The moment density increase is found to have no effect on the average exchange energy between the spin of conduction electrons and localized moments, but promotes an increase of the relaxation time difference between the two spin-dependent conduction channels, leading to the linear decrease of *S* as the Mn content increases. Our observations in model materials coupled with the expression proposed in Eq. (3) open prospects for original engineering routes for the development of spin-engineered thermoelectric materials.

## Materials and methods

Mn_5_Ge_3_ and MnCoGe films were elaborated by magnetron sputtering and solid-state reaction. 99.99% pure Co, 99.99% pure Ge, and 99.9% pure Mn targets were sputtered on 1.5 × 2.5 cm^2^ glass substrates in a commercial magnetron sputtering system with a base vacuum of 10^−8^ Torr^28^. In this system, three targets placed at an angle of 45° to the normal of the sample surface can be simultaneously sputtered during the substrate rotation. Co, Ge, and Mn deposition rates were separately calibrated thanks to the measurement by X-ray reflectivity (XRR) of the thickness of different films deposited in different conditions. The substrates were cleaned 10 min in an acetone bath before to be rinsed 10 min in alcohol in an ultrasonic cleaner. They were finally kept 30 min at 423 K in a baking furnace, before to be loaded in the sputtering chamber. The elements were deposited (Ge and Mn for Mn_5_Ge_3_) or co-deposited (Mn, Co, and Ge for MnCoGe) at room temperature on the glass substrates that were rotated at 5 rpm. The Mn_5_Ge_3_ films were produced by reactive diffusion: 35 nm of Ge were deposited on the glass substrate before to be capped with 31 nm of Mn^30^. After deposition, the samples were ex situ annealed under vacuum (*P* ~ 10^−7^ mbar) at 400 °C for 10 min. The diffractogram (a) in Fig. 2a shows some diffraction peaks belonging to the Ge lattice in addition to the peaks of the phase Mn_5_Ge_3_, meaning that the Ge layer was not entirely consumed by the growth of Mn_5_Ge_3_ despite the entire consumption of the Mn layer. The MnCoGe films were produced by non-diffusive reaction^28^, allowing the stoichiometry of the compound to be varied: Mn, Co, and Ge were co-sputtered on the substrate up to a thickness of 150 nm. The samples were also ex situ annealed at 400 °C for 10 min after deposition.

Sample structure was investigated by both X-ray diffraction (XRD) in the Bragg–Brentano geometry (*θ* − 2*θ*) using a Cu K_α_ source (*λ*_*Kα*_ = 0.154 nm) in a PANalytical X’Pert PRO setup equipped with an X’Celerator detector, and by atomic force microscopy (AFM) using a Solver-PRO system from NT-MDT. Film magnetization was measured versus temperature using a SQUID magnetometer Quantum Design MPMSXL. Hall measurements and sample resistivity were measured in the Van der Pauw geometry using a lab-made setup operating between 20 and 350 K. The applied magnetic field for Hall measurements was 0.5 T. The Seebeck coefficients of the films were measured using a home-made setup^39^ between *T* = 225 and 325 K, close to the FM/PM transition, according to the geometry presented in Fig. 1a. The distance *d* between the two electrodes was 1 cm.

## Supplementary Information

Supplementary Information.

## Data Availability

All data are available in the main text or the supplementary materials.
