# Supplementary material for: Magnetic moment impact on spin-dependent Seebeck coefficient of ferromagnetic thin films
Source: Sci Rep. 2023 Jan 4;13:172. doi: 10.1038/s41598-022-26993-3 (PMC9813267; doi:10.1038/s41598-022-26993-3)
Supplement: Supplementary file 1 — Supplementary Information. [file 41598_2022_26993_MOESM1_ESM.pdf]

Magnetic moment impact on spin-dependent Seebeck coefficient of ferromagnetic thin films

Alain Portavoce[1*], Elie Assaf[1], Maxime Bertoglio[1], Dario Narducci[2], Sylvain Bertaina[1]

[1]*IM2NP, Aix-Marseille University/CNRS, Faculté des Sciences de Saint-Jérôme case 142, 13397 Marseille, France*

[2]*Department of Materials Science, University of Milano-Bicocca, via R. Cozzi 55 - 20125, Milano, Italy*

[*]*Corresponding author. Email: alain.portavoce@im2np.fr*

Supplementary Text

In order to probe the influence of magnetic moments on the spin-dependent *S*, this work focusses on the study of two FM compounds Mn_5Ge_3 and MnCoGe. These two compounds have already been the subject of experimental (32–34,42–43) and theoretical investigations (31,34,41). They can be considered as model FM materials as they both exhibit metallic conductivity (32) and their magnetization is mainly depending on magnetic moments carried by a single element, the Mn atoms in the two cases (31,41). Furthermore, their Curie temperatures are close to RT (32–33, 42–43), allowing the concurrent variations of magnetization and spin-dependent *S* to be investigated close to the ferromagnetic-paramagnetic (FM/PM) transition.

Mn_5Ge_3 is a stoichiometric compound with a hexagonal atomic lattice (Fig. S1a) and a Curie temperature T_c ~ 297 K (32–33). Bulk Mn_5Ge_3 shows a uniaxial magneto-crystalline anisotropy along the c axis of the hexagonal atomic lattice, resulting in an easy axis of magnetization aligned along the crystallographic direction [001] (29). The uniaxial anisotropy constant was found to be comprised between 2.78×10^4 and 3.34×10^4 J m^{-3}. Mn_5Ge_3 shows a metallic behavior in both the minority and majority spin components, and magnetic moments are carried by two different types of Mn ions, noted Mn_I (μ_I ~ $2.1\mu_B$) and Mn_{II} (average μ_I ~ $3.2\mu_B$) in Fig. S1a (31), all the moments being oriented along the crystallographic axis c (30–31). Mn_5Ge_3 thin film properties (conductivity (32), magnetization (33,44), and FM resonance (44)) were characterized in numerous works, studying possible spin-polarized electron injection in Ge for spintronic applications (31–34). In contrast to bulk Mn_5Ge_3, epitaxial Mn_5Ge_3 films of thickness smaller than 50 nm exhibit an easy axis of magnetization lying in the hexagonal basal (001) plane, parallel to the interface between the Mn_5Ge_3 films and the substrate. However, the easy axis of magnetization turns progressively out of this plane for thicknesses above 50 nm, but never becomes perpendicular to the sample surface even for Mn_5Ge_3 films with a thickness larger than 200 nm (33). Spin-polarization of diffusive currents in Mn_5Ge_3 is expected to be larger than 40 % (31–32). Experiments shown that reactive diffusion guaranty the growth of carbon-free stoichiometric Mn_5Ge_3 thin films, allowing a very good reproducibility of film properties (44,49). Consequently, Mn_5Ge_3 thin films were used to investigate the influence of

localized magnetic moment fluctuation with temperature, as well as the influence of moment ordering using external magnetic field on the spin-dependent *S*.

MnCoGe is a ternary compound generally studied for its magnetocaloric properties. MnCoGe thin films have been considerably less studied than Mn_5Ge_3 (42−43). Nevertheless, MnCoGe thin film structural and magnetic properties have been reported in the literature (42−43). Bulk stoichiometric MnCoGe transforms from a low-temperature TiNiSi-type orthorhombic structure (space group Pnma) to a Ni_2In-type hexagonal structure (space group P63/mmc). The transition temperature is comprised between 420 K and 650 K (35). The FM/PM transition occurs at T_c ~ 355 K for the orthorhombic martensitic phase and at T_c ~ 275 K for the hexagonal austenitic phase (39−40). Saturation magnetization M_S is different for the two phases, with M_S = 4.13 μ_B for the orthorhombic phase (37) and M_S = 2.76 μ_B for the hexagonal phase (38). The magnetic moments are localized on both Mn and Co atoms in the orthorhombic phase, while the moments are only carried by the Mn atoms in the hexagonal phase (36).

Thin films elaborated by non-diffusive reaction allow the hexagonal structure shown in Fig. S1b to be stabilized at RT (42). In this case, the Curie temperature of the compound hexa-MnCoGe is T_c ~ 269 K (42). Ab initio calculations (41) show that MnCoGe electronic conduction in hexa-MnCoGe is supported by electronic states close to the Fermi level primarily located on Mn atoms. Furthermore, the magnetic moment of hexa-MnCoGe is mostly carried by Mn atoms (single type of Mn with μ_I ~ 3.09μ_B, Fig. S1b). Consequently, a reduced content of Mn atoms leads to a reduced absolute magnetic moment. T_c is predicted to decrease with Mn concentration, and the magnetic moment to linearly increase with the Mn-Mn distance of nearest neighbors. Indeed, non-diffusive reaction allowed the Mn concentration in hexa-MnCoGe thin films to be increased up to ~ 3 at%. Consequently, hexa-MnCoGe thin films were used to investigate the influence of magnetic moment density on the spin-dependent *S*.

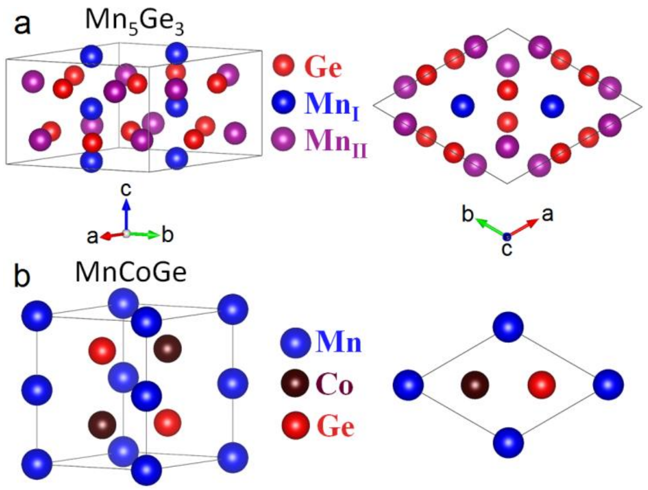

Fig. S1. Crystal structures of Mn_5Ge_3 (**a**) and hexagonal-MnCoGe (**b**) compounds.

S measurements were interpreted using eq. 2 which was derived considering the band diagram model presented in Fig. 1c. Starting from the expressions of the Peltier coefficient Π and the Seebeck coefficient *S* (47)

$$\Pi = -\frac{1}{e}\int [E - E_F + D(E,T)]\frac{\sigma(E)}{\sigma}dE$$
$$S = \frac{\Pi}{T}$$

with e the electron charge, σ the electronic conductivity, E_F the Fermi level, and $D(E,T)$ the additional term taking into account charge carrier flux interactions with phonons, ions or localized magnetic moment in our case; and considering the case of electron conduction i) in a single electronic band and ii) with an electron density low enough to support the use of the Maxwell-Boltzmann statistic, the Seebeck coefficient in each spin-channel can be expressed as:

$$S = \pm\frac{1}{eT}[E - E_F + Rk_BT + D(E,T)]$$

This expression is the usual equation used to describe the Seebeck coefficient of non-degenerated semiconductors. *R* is generally temperature independent (47). It contains both the thermal energy and the scattering effect, assuming that the scattering of charge carriers is elastic, corresponding to the relaxation time expression $\tau = aE^s$, where *E* is the average electron energy, and *s* and a are constants depending on scattering mechanism types. The term $\mu = [E - E_F + Rk_BT + D(E,T)]$ corresponds to the average energy of charge carriers in the electronic flux. The electronic flux resulting from the difference of two opposite fluxes above and below E_F in the case of *S*, two state densities were considered: $N = N^+ + N^-$ for $E > E_F$ and $n = n^+ + n^-$ for $E < E_F$ (+ for spin-up, and − for spin-down, see Fig. 1c). According to Fig. 1c, the electrochemical potential of spin-up electrons ($^+$) was thus expressed as

$\mu_1^+ = \frac{N^+}{N}[(E_F + dE - E_c^+) - E_{eI}^+ + R^+k_BT]$ above E_F,

and $\mu_2^+ = \frac{n^+}{n}[(E_F - dE - E_c^+) - E_{eI}^+ + R^+k_BT]$ below E_F,

with $\Delta\mu^+ = \mu_1^+ - \mu_2^+$ due to the difference of fluxes below and above E_F.

The same considerations for spin-down electrons ($\Delta\mu^- = \mu_1^- - \mu_2^-$), lead to

$\Delta\mu = \Delta\mu^+ + \Delta\mu^- = 2dE + \left(\frac{N^+}{N} - \frac{n^+}{n}\right)(\Delta E_C + \Delta E_{eI} + \Delta Rk_BT)$ in eq. 1.

Neglecting *dE*, one can write

$$\Delta\mu = \left(\frac{N^+}{N} - \frac{n^+}{n}\right)(\Delta E_C + \Delta E_{eI} + \Delta Rk_BT) = \left(\frac{n^-}{n} - \frac{N^-}{N}\right)(\Delta E_C + \Delta E_{eI} + \Delta Rk_BT)$$

And thus,

$$2\Delta\mu = \left[\left(\frac{N^+}{N} - \frac{n^+}{n}\right) - \left(\frac{N^-}{N} - \frac{n^-}{n}\right)\right](\Delta E_C + \Delta E_{eI} + \Delta R k_B T)$$

$\left(\frac{N^+}{N} - \frac{n^+}{n}\right)$ is the proportion difference between higher energy spin-up electrons diffusing toward the cold side of the sample and lower energy spin-up electrons diffusing towards the hot side of the sample (Fig. 1c). This term depends on the DOS asymmetry between spin-up electrons at $E = E_F + d\varepsilon$ and spin-up electrons at $E = E_F - d\varepsilon$. Considering that

$$\left(\frac{dg^+}{d\varepsilon}\right)_{E_F} = \frac{(N^+ - n^+)}{d\epsilon} \text{ and } \left(\frac{dg^-}{d\varepsilon}\right)_{E_F} = \frac{(N^- - n^-)}{d\epsilon}$$

one can write

$$N - n = \left(\frac{dg^+}{d\varepsilon}\right)_{E_F} d\varepsilon + \left(\frac{dg^-}{d\varepsilon}\right)_{E_F} d\varepsilon \text{ and } N^+ = \left(\frac{dg^+}{d\epsilon}\right) d\varepsilon + n^+$$

Giving

$$\frac{N^+}{N} - \frac{n^+}{n} = \frac{\left(\frac{dg^+}{d\varepsilon}\right)_{E_F} d\varepsilon + n^+}{\left(\frac{dg^+}{d\varepsilon}\right)_{E_F} d\epsilon + \left(\frac{dg^-}{d\varepsilon}\right)_{E_F} d\epsilon + n} - \frac{n^+}{n}$$

Assuming $\left(\frac{dg^+}{d\varepsilon}\right)_{E_F} d\epsilon + \left(\frac{dg^-}{d\varepsilon}\right)_{E_F} d\epsilon \ll n$ (see Fig. 1c)

$$\frac{N^+}{N} - \frac{n^+}{n} \sim \frac{\left(\frac{dg^+}{d\varepsilon}\right)_{E_F} d\varepsilon + n^+}{n} - \frac{n^+}{n} = \frac{1}{n}\left(\frac{dg^+}{d\varepsilon}\right)_{E_F} d\varepsilon$$

And

$$\frac{N^-}{N} - \frac{n^-}{n} \sim \frac{1}{n}\left(\frac{dg^-}{d\varepsilon}\right)_{E_F} d\varepsilon$$

Leading finally to

$$\Delta\mu = \frac{1}{2}\frac{d\varepsilon}{n}\left[\left(\frac{dg^{+}}{d\epsilon}\right)_{E_F} - \left(\frac{dg^{-}}{d\epsilon}\right)_{E_F}\right](\Delta E_C + \Delta E_{eI} + \Delta R k_B T)$$

And to the Seebeck coefficient expression (eq. 2)

$$S = -\frac{1}{2eT}\frac{d\varepsilon}{n}\left[\left(\frac{dg^{+}}{d\epsilon}\right)_{E_F} - \left(\frac{dg^{-}}{d\epsilon}\right)_{E_F}\right](\Delta E_C + \Delta E_{eI} + \Delta R k_B T)$$
